# Artificial intelligence in higher education and graduate employability: cognitive engagement, self-efficacy, and career adaptability in the AI era

**DOI:** 10.3389/fpsyg.2026.1987347

**Published:** 2026-10-29

**Authors:** Lingyuan Kong, Honghui Zhang

**Affiliations:** Student and Graduate Affairs Department, Shanghai University of Political Science and Law, Shanghai, China

**Keywords:** artificial intelligence, career adaptability, cognitive engagement, graduate employability, higher education, self-efficacy, self-regulated learning

## Abstract

Artificial intelligence (AI), particularly generative AI, is increasingly used in higher education, but its contribution to graduate employability cannot be inferred from access to AI or frequency of use alone. This critical narrative review examines how AI-supported learning may relate to employability through processes discussed in educational psychology and career development. Searches of Web of Science, Scopus, ERIC, and PubMed covered database inception to 1 July 2026, with supplementary searches completed on 2 October 2026. The literature was synthesized interpretively and critically, with attention to the type of outcome measured and the extent to which findings support the proposed relationships. The synthesis focuses on cognitive engagement and self-regulated learning, self-efficacy and motivational regulation, and career adaptability. Three tensions organize the discussion: capability augmentation and cognitive offloading, career agency and AI-related insecurity, and resource access and capability differences. Included studies report associations or short-term changes in outcomes such as engagement, AI literacy, self-efficacy, career adaptability, and perceived employability. Direct evidence linking AI-supported learning to later employment outcomes remains limited. The proposed framework should therefore be read as a set of theory-informed pathways for future testing, rather than as an established causal or temporal sequence. Implications for assessment, disciplinary AI literacy, career education, institutional support, and future research are discussed.

## 1 Introduction

Artificial Intelligence (AI), especially generative AI, is changing how university students search for information, receive explanations, draft and revise work, solve problems, and prepare for careers. These changes are often framed as questions of technological adoption or digital skill. A more consequential educational question concerns what AI changes in the learner: whether students remain cognitively engaged, regulate their learning, develop realistic beliefs about their competence, and use learning experiences to adapt to an uncertain future. Graduate employability provides an important distal outcome for this question because the value of higher education depends not only on what students can produce with technological assistance, but also on what they understand, can judge, can transfer, and can continue to develop after graduation.

Graduate employability is therefore treated here as a developmental rather than purely labor-market construct. Employability research has long moved beyond a checklist of disciplinary knowledge and generic skills toward relational and capital-based accounts that incorporate identity, psychological resources, adaptability, and the capacity to mobilize learning across contexts (Fugate et al., 2004; Tomlinson, 2012; Eimer and Bohndick, 2023). AI intensifies this shift because it changes both capability formation and the environment in which capabilities are evaluated. AI can extend access to feedback, information, rehearsal, and task support, while also making it easier to bypass the cognitive activity through which durable capability develops. It can increase confidence and career exploration, while simultaneously heightening perceptions of replaceability and uncertainty. It can widen access to educational resources, while differences in prior knowledge, AI literacy, institutional support, and access to high-quality tools may produce unequal learning gains (Beckman et al., 2025; Drydakis, 2025).

Educational psychology provides a useful framework for explaining why AI-supported learning can produce both beneficial and adverse outcomes. Cognitive engagement reflects the extent to which students invest effort in analyzing, evaluating, and constructing meaning during learning, and recent meta-analytic evidence suggests that generative AI can enhance cognitive engagement when it is used to support active learning, although over-reliance may produce disengagement and shallow processing (Heung and Chiu, 2025). Self-regulated learning further captures learners' capacity to plan, monitor, evaluate, and adapt their learning strategies, processes that remain particularly important when students interact with AI systems that can readily perform parts of a task on their behalf (Alhazbi et al., 2024; Luo and Zhou, 2024). Self-efficacy represents students' perceived capability to manage academic demands and use available resources effectively; emerging evidence in higher education indicates that perceived AI capability, independent learning, and academic self-efficacy are closely interconnected (Cheung et al., 2025). Career adaptability extends these learning-related resources into the transition from university to work by enabling students to anticipate change, maintain a sense of control, explore alternative pathways, and sustain confidence under occupational uncertainty (Zhao et al., 2024; Wang, 2026). Taken together, these constructs suggest that the relationship between AI use and graduate employability is better understood as a developmental learning-to-employability pathway, in which the educational value of AI depends on whether its use strengthens cognitive engagement, self-regulation, efficacy beliefs, and adaptive career resources rather than merely improving task efficiency.

Existing reviews have provided strong syntheses of AI in higher education, AI literacy, employability, and institutional implementation, but the literatures remain fragmented across learning science, career psychology, graduate-capital perspectives, and labor-market change (Almatrafi et al., 2024; Bond et al., 2024; Jung et al., 2024; Wang P. et al., 2025). The contribution of the present review is not another thematic inventory of benefits and risks. It integrates three recurrent tensions-augmentation vs. cognitive offloading, career agency vs. AI-related insecurity, and resource democratization vs. capability stratification-with three educational-psychological mechanisms: cognitive engagement and self-regulation, self-efficacy and motivational regulation, and career adaptability. The review focuses on (1) how AI-supported learning changes these psychological processes, (2) how they help explain variation in graduate-employability outcomes, and (3) under what educational and contextual conditions AI-related resources are converted into sustainable capability rather than superficial performance.

## 2 Theoretical background

### 2.1 Graduate employability and AI literacy

Graduate employability refers to the developing capacity to secure, sustain, and progress in work by mobilizing personal resources within social, institutional, and labor-market contexts. It is therefore distinct from employment status alone (Tomlinson, 2012; Holmes, 2013; Eimer and Bohndick, 2023). In this review, AI literacy refers to the ability to understand AI capabilities and limitations, use AI appropriately, evaluate its outputs, and act responsibly (Almatrafi et al., 2024; Hackl et al., 2026). AI literacy may be relevant to employability when students use disciplinary knowledge to assess whether AI-generated information is accurate, appropriate, and professionally relevant. This positions AI literacy as a potential resource for learning and work; it does not establish that AI literacy leads to employment or better employment outcomes.

### 2.2 AI-supported learning, cognitive engagement, and offloading

Cognitive engagement refers to the mental effort learners invest in understanding and evaluating material (Heung and Chiu, 2025). Self-regulated learning involves planning, monitoring, and adjusting one's learning (Alhazbi et al., 2024). These processes are relevant to AI-supported learning because AI may change how learners distribute effort between themselves and the tool. Cognitive offloading occurs when mental work is delegated to an external resource. AI may reduce task demands, but learning may be hindered when it replaces learners' reasoning or checking. The implications depend on which tasks are delegated and whether learners evaluate the output (Tian and Zhang, 2025; He et al., 2026). Travasi (2025) distinguishes automation from augmentation and discusses the potential complementarity of human intellect and AI. Applied to education, this perspective draws attention to whether students remain responsible for interpreting information, evaluating outputs, and making decisions. It provides a conceptual lens for this review, rather than evidence of improved graduate employment.

### 2.3 Psychological and career-related resources

Academic self-efficacy refers to students' beliefs about their ability to complete academic tasks. AI self-efficacy concerns their perceived ability to use, understand, and critically evaluate AI. Both are beliefs about capability and should not be treated as evidence of demonstrated skill (Acosta-Enriquez et al., 2025; Bećirović et al., 2025). Motivational regulation refers to strategies for sustaining effort when motivation declines and is closely related to self-regulated learning (Villar et al., 2024).

Career adaptability comprises psychosocial resources for responding to career tasks, transitions, and uncertainty. Its dimensions are concern, control, curiosity, and confidence. Career adaptability is a career-development resource, not an employment outcome (Savickas and Porfeli, 2012; Khampirat, 2024). The term career adaptability scale is used only when referring to the specific instrument.

### 2.4 Institutional conditions and conceptual scope

The translation of AI-related resources into learning and career development may also depend on institutional conditions. In a study of administrative staff at Italian public universities, Travasi et al. (2026) examined human and relational barriers to AI-related institutional change, including issues concerning autonomy, competence, and relatedness (Travasi et al., 2026). As that study concerns university employees rather than students, it informs the institutional context of the framework only; it is not direct evidence about student learning or graduate employability. Together, these concepts inform a proposed account of how AI-supported learning may relate to graduate employability through learning, psychological, and career-development processes. Figure 1 presents this conceptual account. The relationships shown are theoretical propositions and should not be interpreted as an established causal or temporal sequence.

**Figure 1:**
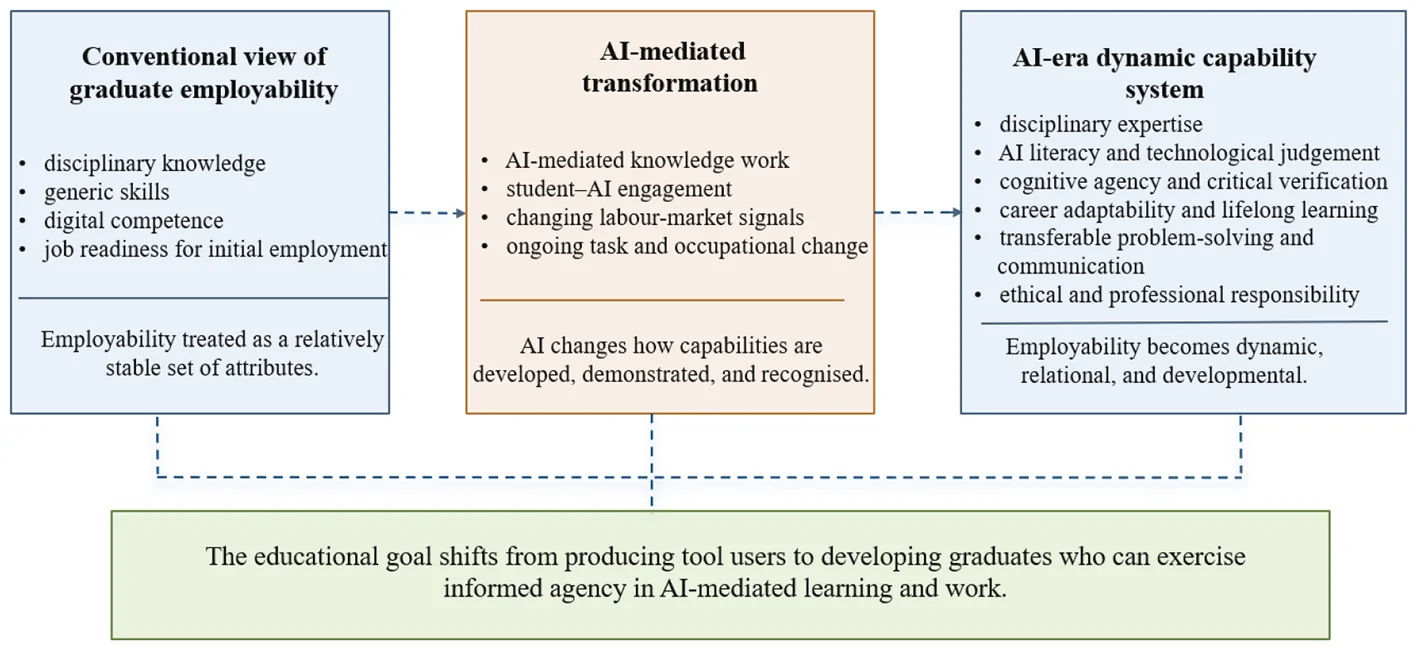
Conceptual transition from a static employability view to a dynamic capability system in the AI era.

## 3 Methodology

### 3.1 Review design

This study adopted a critical narrative review to integrate research on the use of artificial intelligence in higher education and graduate employability, with a focus on the relationships among learning processes, psychological mechanisms, and career development. Given the interdisciplinary nature of the topic and the range of outcomes considered, we used an interpretive narrative synthesis rather than quantitative pooling. Through iterative reading of the relevant literature and theories, we identified three tensions: capability augmentation vs. cognitive offloading, career agency vs. AI-related insecurity, and resource democratization vs. capability stratification. These tensions informed the development of the proposed theoretical framework.

### 3.2 Literature search and study selection

Web of Science, Scopus, ERIC, and PubMed were searched. The main search covered each database from its inception to 1 July 2026, with supplementary searches conducted through 2 October 2026. Priority was given to AI-related studies published in 2023 or later. Reference lists of relevant publications were also consulted to identify foundational theoretical work. The following Boolean search string was used across the databases, (“artificial intelligence” OR “generative AI” OR “generative artificial intelligence” OR ChatGPT OR “large language model” OR “AI literacy” OR “AI adoption”) AND (“higher education” OR university OR universities OR college OR colleges OR undergraduate OR undergraduates OR “graduate students”) AND (employability OR employment OR “career readiness” OR “career adaptability” OR “career development” OR “career guidance” OR “career decision making” OR “career commitment” OR “self-efficacy” OR “self-regulated learning” OR “cognitive engagement” OR “student engagement” OR motivation OR anxiety OR “perceived employability”).

All retrieved records were imported into EndNote for deduplication and then screened by title, abstract, and full text. Two authors (LYK and HHZ) conducted the screening, and disagreements were resolved through discussion. The final corpus comprised 46 publications; the selection process is summarized in Figure 2.

**Figure 2:**
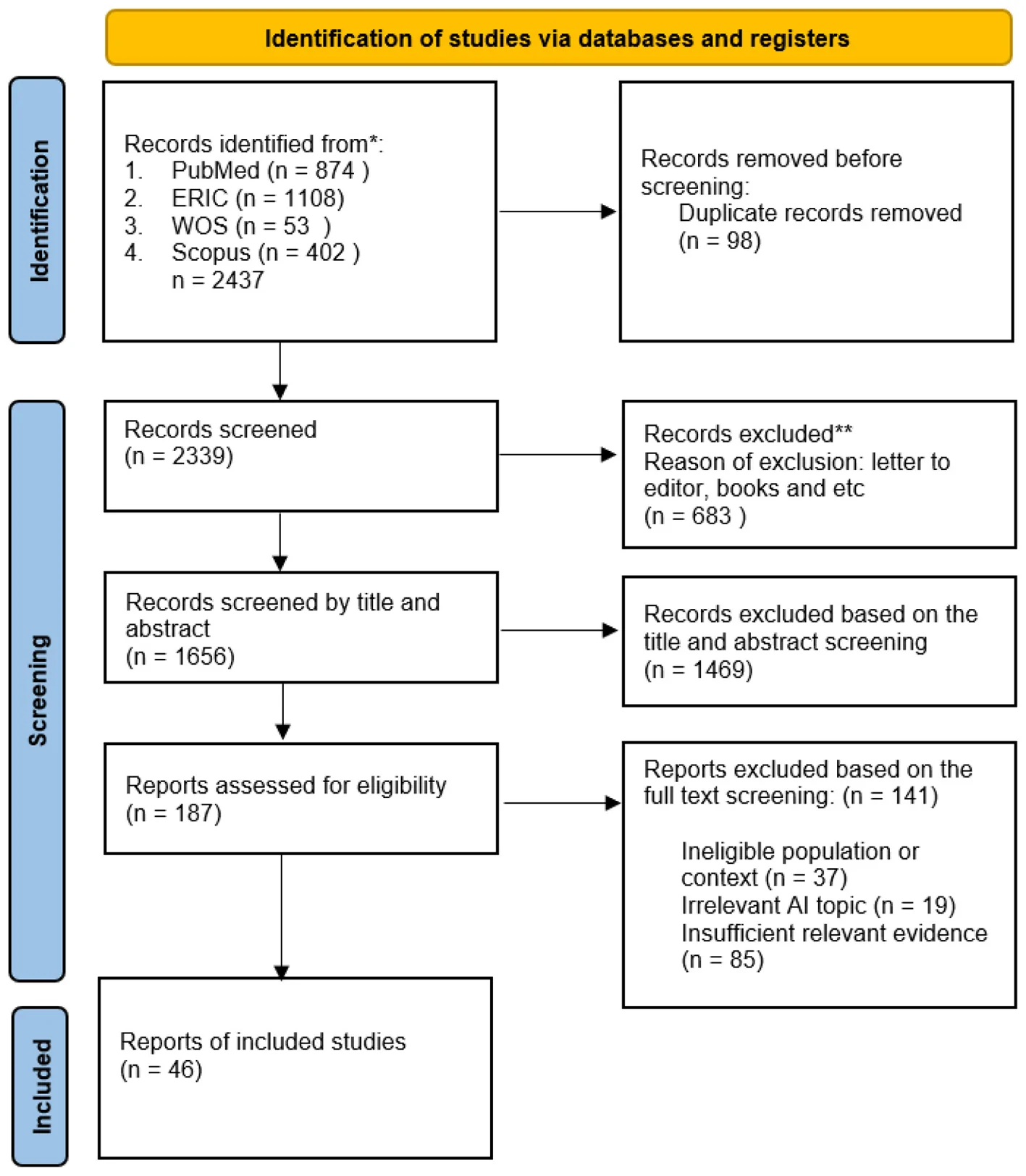
Flow diagram of literature identification and selection.

The literature was read and compared iteratively in relation to the review questions, and themes were developed through this interpretive process. Evidence was first organized by the core constructs addressed, AI application context, study populations and disciplines, research designs, and outcome types. Recurring relationships, divergent findings, and contextual conditions were then compared across studies, leading to the development of five themes. For each theme, the number and types of relevant publications, study settings and populations, areas of convergence and divergence, and limitations related to study design are described. Methodological strengths and limitations are discussed descriptively, with consideration of study design, sampling, measurement, and follow-up. The synthesis also distinguishes between study populations and levels of analysis. Studies involving higher education students or graduates inform the discussion of individual-level learning, psychological, and career-related outcomes. Studies involving university faculty or staff, as well as institutional- and labor-market-level research, are considered evidence about the educational and institutional context. Findings from different populations and levels are not treated as equivalent.

### 3.3 Inclusion and exclusion criteria

English-language publications were considered if they addressed AI-related learning, psychological, career, or employment processes in higher education. Studies of students and recent graduates formed the core evidence; studies involving university staff, employers, institutions, or labor markets were included only as context relevant to graduate employability. Eligible sources included empirical studies, reviews, theoretical or conceptual papers, and relevant foundational literature. AI-related studies published from 2023 onward were prioritized, while foundational work could predate this period. Studies without substantive relevance and those focused solely on AI technical performance were excluded (Table 1).

**Table 1:** Inclusion and exclusion criteria.

Inclusion criteria	Exclusion criteria
Higher education students or recent graduates; relevant staff, institutional, employer, or labor-market studies used as context.	Primary or secondary education studies without a clear link to the review; contextual studies unrelated to the review questions.
AI-related learning, teaching, assessment, literacy, or career preparation in higher education.	AI technical studies without an educational or career-related focus.
At least one relevant learning, psychological, career, employment, or contextual construct.	AI is incidental, or no findings relevant to the review questions are reported.
Relevant empirical studies, reviews, theoretical or foundational works, and contextual sources.	Editorials, letters, book reviews, or abstracts lacking substantive information.
English-language publications identified by 2 October 2026; AI studies from 2023 onward were prioritized, while earlier foundational works were eligible.	Non-English, unverifiable, or insufficiently detailed sources.

## 4 Evidence on Artificial Intelligence and Graduate Employability

To distinguish among outcomes measured at different levels, the literature was grouped into four categories. Direct employment outcomes included employment status, time to employment, job quality, income, retention, and employer evaluations. Indirect career indicators included perceived employability, career readiness, career exploration, career decision-making, and career adaptability. These indicators reflect students' perceptions or resources relevant to career development, but do not directly measure whether graduates obtain jobs, secure higher-quality work, or perform effectively in the workplace. Proximal learning and psychological outcomes included AI literacy, engagement, self-regulated learning, self-efficacy, motivation, anxiety, and course-related performance. The fourth category comprised theoretical propositions concerning how AI-supported learning might relate to career development or employment through learning and psychological processes. Studies involving university employees, institutional policies, or implementation were treated as contextual evidence rather than direct evidence of student-level learning or graduate employment. Claims about employment outcomes are based only on studies that measured such outcomes directly. The characteristics of the included studies, including their designs, AI application contexts, and primary outcomes, are presented in Table 2. Table 3 summarizes how the themes relate to the proposed pathways.

**Table 2:** Characteristics and principal findings of the included studies.

References	Country, discipline and sample	Design and AI context	Outcome level and principal findings
Yang S.-H. et al. (2025)	China, Taiwan; hospitality management and computer-related disciplines; students, *n* = 66	Quasi-experimental study of GenAI-assisted collaborative drama-based role-play in a metaverse learning environment	Proximal learning outcomes. Learning achievement, engagement, professional identity and related skills improved; no significant between-group difference was found for accountability.
Hendrawan and Lestari (2026)	Indonesia; management; final-year university students, *n* = 455	Cross-sectional survey using PLS-SEM; AI literacy, career adaptability and growth mindset	Indirect career outcomes. AI literacy was positively associated with career adaptability and perceived employability. The statistical mediation model does not establish causality.
Singh et al. (2026)	Saudi Arabia; business students; valid sample, *n* = 652	Cross-sectional survey of AI-based learning tools and student engagement	Proximal and indirect outcomes. The study examined associations among AI tool use, engagement, academic performance, perceived employability and empowerment. The sample came from one university.
Song et al. (2025)	China; nursing undergraduates; 144 initially recruited, 132 analyzed	Quasi-experimental study of GenAI-supported problem-based nursing instruction	Proximal learning outcomes. The study examined higher-order thinking and AI literacy and reported potential benefits of structured instructional activities.
Testa et al. (2026)	Italy; business, economics and management students; *n* = 339	Cross-sectional survey of AI literacy, AI readiness and AI self-efficacy	Indirect career outcomes. AI readiness was statistically associated with AI literacy and career adaptability. The design cannot establish temporal order or causality.
Segbenya et al. (2023)	Ghana; postgraduate students; *n* = 294	Cross-sectional survey of factors associated with AI use	Proximal skills outcomes. The study examined the relationship between AI use and 21st-century employability skills; it did not measure actual employment.
Drydakis (2025)	United Kingdom; non-STEM university students; *n* = 120	Twelve-week AI module, pre- and post-course assessment of AI capital, and employment follow-up six months after graduation	Direct employment outcome. AI capital increased after the course and was associated with later employment status. The single-course, non-randomized design does not establish that the course caused improved employment.
Li M. et al. (2025)	Thailand; International Chinese Language Education graduates, career counselors and HR professionals; *n* = 30	Qualitative interviews on graduate employability barriers and institutional support in the AI era	Career and institutional context. Participants identified curriculum gaps in AI skills, fragmented skills development and insufficient institutional support. Views from different participant groups should not be treated as individual student outcomes.
Li R. et al. (2025)	China; university students; *n* = 455	Cross-sectional survey and structural equation modeling of career self-efficacy, AI attitudes, AI literacy and job-seeking anxiety	Proximal psychological outcomes. Career self-efficacy, AI attitudes and AI literacy were statistically associated with job-seeking anxiety. Subsequent job-search or employment outcomes were not tracked.
Qi and Feng (2025)	China; applied university students and institutional programmes; reported sample, *n* = 300	Institutional-level industry–education integration model using a back-propagation neural network	Institutional employment outcome, included as contextual evidence. The study reported higher employment rates and several competency measures in the experimental group. It did not directly examine student learning or psychological mechanisms related to generative AI use.
Duan et al. (2026)	China; university students; *n* = 315	Cross-sectional survey and structural equation modeling of AI anxiety, career adaptability, self-efficacy and career decision-making	Indirect career outcomes. AI anxiety was negatively associated with career decision-making, with career adaptability showing a statistical mediation effect. The moderating effect of self-efficacy was not supported.
Zheng et al. (2026)	China; undergraduates from four universities; *n* = 411	Cross-sectional survey and structural equation modeling of AI-supported learning experience	Indirect career outcomes. AI-supported learning experience was associated with innovation ability and career adaptability. The cross-sectional mediation model does not establish causal order.
Zhang J. et al. (2026)	China; students from ten universities; valid sample, *n* = 460	Survey of AI awareness and career education	Indirect career outcomes. The study examined statistical relationships among AI awareness, career education, self-efficacy, employability confidence and perceived employability. Actual employment was not followed.
Yang and Li (2025)	China; university graduate employment data; sample and data sources are not clearly reported	Educational data mining and machine-learning analyses of AI and graduate employment	Contextual evidence. The study discusses AI and changes in employment patterns. It should not be treated as core evidence for student learning mechanisms or causal employment effects.
Wei et al. (2026)	China; 770 valid final-year students aged 21–23. The fields included accounting, business administration and international trade. A total of 812 questionnaires were collected, of which 770 were valid.	Cross-sectional survey of confidence in generative AI and perceived employability	Indirect career outcomes. The study examined associations between AI confidence and perceived employability. Some mediation pathways were not significant, and outcomes were self-reported.
Shi and Wang (2025)	Hangzhou, China; university graduates; *n* = 42	Quasi-experimental study of short-term AI mock-interview training	Indirect career outcomes. The study reported changes in perceived employability and simulated interview performance. Simulated interview performance is not equivalent to actual hiring outcomes.
Xu et al. (2025)	China; university students; analyzed sample, *n* = 68	Quasi-experimental comparison of generative AI learning with and without metacognitive prompts	Proximal learning outcomes. Some self-regulated learning and learning experience measures improved. The between-group difference in academic performance was not statistically significant.
Parveen and Alkudsi (2024)	Saudi Arabia; undergraduate and master's students across disciplines; *n* = 465	Survey of student views on AI integration, future skills and career readiness	Indirect career outcomes. The findings primarily reflect perceptions of future skills and career readiness; actual employment was not measured.
Li et al. (2026)	China; undergraduate career-planning documents; 36 documents	Text-mining analysis of career-planning documents in the generative AI era	Indirect career outcomes. The study identified career-readiness and adaptation profiles. Planning documents do not demonstrate actual career behavior or employment outcomes.
Yuqian et al. (2026)	China; vocational college students; valid sample, *n* = 2,622	Cross-sectional survey using latent profile and learning-path analyses of generative AI learning support	Indirect career outcomes. The direct association between AI learning support and employability was not significant. The reported relationships were mainly through interdisciplinary motivation or ability.
Kannangara and Yatigammana (2026)	Sri Lanka; undergraduates; *n* = 74	Exploratory experiment comparing ChatGPT-assisted problem-solving with traditional instruction	Proximal learning outcomes. Problem-solving performance was higher in the ChatGPT group. Career readiness and actual employment were not measured.
Wut et al. (2025)	Hong Kong, China; university students; *n* = 208	Cross-sectional survey using PLS-SEM to examine AI literacy, AI attitudes and perceived employability	Indirect career outcomes. AI literacy was associated with internal and external perceived employability. No later employment follow-up was conducted.
Rook and Plumb (2026)	Australia; undergraduate business students; *n* = 166	Qualitative survey of student views on integrating generative AI into curricula and career preparation	Indirect career outcomes. Students supported curriculum content on AI capabilities, applications and ethics. The findings reflect student views, not measured changes in capability or employment.
Semenikhina and Drushlyak (2026)	Ukraine; university students; *n* = 354	Ukrainian subsample of an international student survey on perceived opportunities and threats of ChatGPT	Indirect career outcomes. The study describes students' perceived career opportunities and threats related to AI, rather than actual employment outcomes.
Algül and Yarbaşi (2026)	Türkiye; university students across disciplines; *n* = 500	Cross-sectional survey of AI awareness and employment expectations	Indirect career outcomes. Dimensions of AI awareness were positively associated with perceived employability. The cross-sectional design cannot determine direction of association.
Xiao and Zheng (2025)	Multinational student survey; analytical sample approximately *n* = 9,384	Cross-sectional analysis of student ChatGPT use and employment confidence	Indirect career outcomes. The association between ChatGPT use and employment confidence was stronger among undergraduates and social science students. Confidence is not equivalent to employment rates.
Dahri et al. (2025)	Malaysia; university students; *n* = 290	Cross-sectional survey and structural equation modeling of ChatGPT use, cognitive skills and career readiness	Proximal and indirect outcomes. High-quality outputs and personalized learning were associated with cognitive skills and career-related knowledge. Trust and information access did not significantly predict all outcomes.
Zehui et al. (2025)	China; vocational college students; *n* = 697	Cross-sectional survey of transformational leadership, AI literacy and career self-regulation	Indirect career outcomes. AI literacy and career self-regulation were tested as statistical mediators of future work readiness. Employment after graduation was not measured.
Luo et al. (2023)	China; nursing students; *n* = 1,788	Cross-sectional online survey of AI literacy, perceived employability and demand for ChatGPT	Indirect career outcomes. The study describes nursing students' views and demands related to AI and employability; it did not evaluate an AI intervention.
Jiang and Chen (2026)	China; undergraduates; *n* = 576	Cross-sectional survey and structural equation modeling of AI trust, intrinsic motivation, job insecurity and protean career orientation	Indirect career outcomes. The study analyzed associations between AI trust and career orientation, including the roles of intrinsic motivation and job insecurity.
Shao and Wang (2026)	China; STEM undergraduates; *n* = 200	2 × 2 quasi-experiment of DeepSeek-assisted writing, with students grouped by prior academic performance	Proximal psychological outcomes. AI-assisted writing was associated with changes in post-task motivation and self-efficacy, with larger benefits among lower-achieving students. Academic performance was not significant in every between-group comparison.
Shen et al. (2026)	China; students at a comprehensive university; *n* = 689	Cross-sectional survey using PLS-SEM to examine AI acceptance, AI attitudes, career adaptability and perceived employability	Indirect career outcomes. Positive AI attitudes and career adaptability were associated with perceived employability. Statistical mediation does not establish a developmental sequence.
Cantaş et al. (2026)	Northern Cyprus; pre-service teachers; valid sample, *n* = 396	Cross-sectional survey of AI anxiety, 21st-century skills and lifelong learning tendency	Proximal skills outcomes. AI anxiety was associated with some skill domains but did not significantly predict lifelong learning tendency.
Wu and He (2026)	China; undergraduates; *n* = 350	Cross-sectional survey using a protection motivation framework to examine AI anxiety and AI learning intention	Proximal psychological outcomes. The study analyzed relationships among threat perceptions, coping efficacy, anxiety and learning intention; employment outcomes were not measured.
Liang and Du (2026)	China; undergraduate and postgraduate humanities and social science students; valid sample, *n* = 548	Cross-sectional survey of perceived AI threat, controllability and effort–reward imbalance	Proximal psychological outcomes. The study examined psychological factors associated with perceived AI threat; actual employment outcomes were not measured.
Zhang L. et al. (2026)	China; university students and early-career individuals; scenario experiment, *n* = 205, followed by survey, *n* = 458	Two studies examining AI replacement threat and career-goal adjustment	Indirect career outcomes. The study examined associations between AI replacement threat and career-goal adjustment intentions. Career intentions are not equivalent to career behavior or employment outcomes.
Chen and Wang (2026)	China; university students; valid sample, *n* = 564	Multi-method study using structural equation modeling, necessary condition analysis and fuzzy-set analysis	Indirect career outcomes. The study analyzed the relational structure and combinations of conditions associated with career adaptability under AI-related employment pressure.
Myagmarkhorloo et al. (2026)	Mongolia; university students in applied language studies; 267 initially enrolled, 211 analyzed	Four-time-point within-subject longitudinal study, with AI-enhanced flipped learning introduced after standard flipped learning	Proximal learning outcomes. Engagement and self-regulated learning changed over time. The fixed sequence means time and order effects cannot be ruled out.
Aditya et al. (2024)	Palestine, Türkiye and Chile; university students; *n* = 207	Multinational course project in which an AI teacher delivered nine lessons on employability and transferable skills	Proximal and indirect outcomes. The study reported course engagement, completion and satisfaction. There was no control group or employment follow-up.
Portocarrero Ramos et al. (2025)	Peru; undergraduate and postgraduate graduates; valid sample, *n* = 148	Cross-sectional survey of AI skills and graduate employment	Direct employment-related outcome. Employment status was self-reported. The cross-sectional design cannot establish that AI skills caused employment differences.
Cheng et al. (2026)	United States; business analytics students; final sample, *n* = 143	Cross-sectional survey of AI-supported learning, academic interest and career intentions	Indirect career outcomes. Continued intention to use AI did not show a significant direct association with career-choice intention.
Wang K. et al. (2025)	China; university students; *n* = 502	Survey using PLS-SEM and fsQCA to examine generative AI use for career information seeking	Career-preparation behavior. Career information seeking was associated with combinations of personal knowledge, technological readiness and perceptions of information quality.
Martin et al. (2025)	United States; recent engineering graduates; *n* = 14	Qualitative interviews on AI and engineering careers	Graduate career experience, included as contextual evidence. Several participants reported limited formal AI learning and emphasized the need to align curricula with changing industry expectations.
Yaşar and Karagucuk (2025)	Türkiye; university students in English-related disciplines; survey, *n* = 332, interviews, *n* = 9	Explanatory mixed-methods study of AI anxiety and career decidedness	Indirect career outcomes. No significant relationship was found among English language teaching and English language and literature students. A weak negative association was found among translation and interpreting students, suggesting disciplinary variation.
Fifani et al. (2026)	Morocco; human resource master's graduates; *n* = 45	Multi-university qualitative interviews on soft skills and employability in an AI-enabled workplace	Graduate workplace context, included as contextual evidence. Participants highlighted communication, leadership, emotional intelligence, critical thinking and AI literacy. The study did not test an AI-learning intervention in higher education.
Yang Y. et al. (2025)	Anhui, China; undergraduates in two classes; *n* = 61	Twelve-week quasi-experiment comparing a co-regulated AI virtual teacher with standard AI support	Proximal learning outcomes. The study assessed English writing and self-regulated learning; graduate employment and workplace performance were not measured.

**Table 3:** Synthesis matrix linking review themes, proposed pathways, and related findings.

Theme and supporting references	Proposed pathway	Relevant findings from the studies
AI literacy and disciplinary judgement (Ng et al., 2021; Almatrafi et al., 2024; Bećirović et al., 2025; Song et al., 2025; Hackl et al., 2026; Testa et al., 2026)	AI knowledge, tool-use skills, and critical judgement may help students assess AI outputs against disciplinary standards and may be associated with output quality, self-efficacy, and academic performance.	Studies describe AI literacy as a multidimensional construct involving understanding, using, and evaluating AI, as well as ethical awareness. Research reports links among AI literacy, evaluative ability, disciplinary knowledge, output quality, self-efficacy, and academic performance. A nursing education study found that students' AI literacy improved following generative AI-supported teaching, alongside evidence-based professional judgement. Another study reported a statistically significant mediating role for AI readiness in the relationship between AI literacy and career adaptability.
Cognitive engagement and self-regulated learning: augmentation and cognitive offloading (Alhazbi et al., 2024; Heung and Chiu, 2025; Tian and Zhang, 2025; Xu et al., 2025; He et al., 2026; Myagmarkhorloo et al., 2026)	Using AI to compare, verify, and revise ideas while retaining responsibility for independent thinking may support cognitive engagement and self-regulated learning. Delegating analysis and reasoning directly to AI may reduce active cognitive effort.	A meta-analysis found that generative AI use in active learning contexts was associated with greater cognitive engagement. A longitudinal study observed increases over time in student participation and several self-regulated learning strategies during an AI-supported flipped classroom. Another study found higher task-strategy and self-evaluation scores after metacognitive prompts were introduced; however, the between-group difference in task performance was not statistically significant. Research has also reported an association between AI dependence and critical-thinking performance.
Self-efficacy and motivational regulation: career agency and AI-related insecurity (Villar et al., 2024; Acosta-Enriquez et al., 2025; Cheung et al., 2025; Duan et al., 2026; Shao and Wang, 2026; Wang, 2026; Wu et al., 2026; Wu and He, 2026)	AI-supported practice and feedback may help students sustain motivation and form judgements about their capabilities. AI anxiety, dependence, and concerns about occupational replacement may be related to perceived control and willingness to take career action.	Studies report associations among perceived AI capability, academic self-efficacy, and self-directed learning. Research on AI-assisted writing found that students' intrinsic motivation and writing self-efficacy varied with learning conditions and prior academic performance. Other studies examined relationships between AI anxiety and career decision-making, career adaptability, and AI dependence.
Career adaptability: a psychological resource linking learning and career development (Savickas and Porfeli, 2012; Bond et al., 2024; Zhao et al., 2024; Shen et al., 2026; Testa et al., 2026; Wang, 2026)	AI literacy, attitudes toward AI, and AI self-efficacy may be associated with career adaptability. Career adaptability may, in turn, be related to career exploration, career decision-making, and perceived employability.	Studies report statistical associations among AI acceptance, positive attitudes toward AI, and career adaptability. Another study found that the relationship between AI literacy and career adaptability involved AI readiness and AI self-efficacy. Longitudinal research on careers has also reported associations between career adaptability, career commitment, career identity, and career wellbeing.
Equity and institutional conditions: resource access and differences in capability (Beckman et al., 2025; Drydakis, 2025; Ni and Li, 2026; Shao and Wang, 2026)	Access to AI tools and learning guidance, students' existing disciplinary knowledge and digital capabilities, and institutional factors such as assessment, teacher support, and governance may shape how AI resources are used in learning and career development.	Studies have examined differences among university students in generative AI use and access to resources. Empirical findings indicate that motivation and self-efficacy outcomes in AI-supported learning may vary with students' prior academic performance. Reviews have also identified equity, ethics, collaboration, and the quality of educational implementation as important issues in the use of AI in higher education.

### 4.1 AI Literacy and disciplinary judgement: resources relevant to employability

Evidence for this theme comes primarily from conceptual frameworks and review studies of AI literacy, including discussions of its dimensions and assessment (Ng et al., 2021; Almatrafi et al., 2024; Hackl et al., 2026). These studies generally describe AI literacy as encompassing more than the ability to operate AI tools. It also involves understanding AI capabilities and limitations, evaluating AI-generated outputs, using AI systems appropriately, and acting responsibly. The literature further emphasizes the role of disciplinary knowledge: students need to apply standards from their field to judge whether AI-generated information is relevant, accurate, and professionally appropriate.

These findings support viewing AI literacy and disciplinary judgement as potential resources relevant to employability. However, current evidence focuses mainly on conceptual definitions, educational competencies, and AI-related learning, it does not establish that higher AI literacy leads to higher employment rates, better job quality, or stronger workplace performance. Whether these capabilities translate into an advantage in recruitment or professional practice remains to be tested.

### 4.2 Cognitive engagement and self-regulated learning in AI-supported learning

Research in this area includes reviews, cross-sectional studies, quasi-experimental studies, and longitudinal studies. A within-subjects longitudinal study found that student engagement and self-regulated learning increased during the AI-enhanced flipped-classroom phase, particularly agentic engagement and metacognitive regulation (Myagmarkhorloo et al., 2026). A quasi-experimental study in nursing education and other studies involving university students reported that structured AI activities may support higher-order thinking, AI literacy, task strategies, and self-evaluation (Song et al., 2025; Xu et al., 2025). In the study by Xu et al., 68 university students participated, but the difference in academic performance between groups was not statistically significant.

The findings point to the importance of task design. When students first develop their own ideas, evaluate AI-generated outputs, and explain their revisions, AI may support engagement and self-regulated learning. AI dependence, however, has been associated with weaker critical-thinking performance, and cognitive fatigue has been proposed as one possible explanation (Tian and Zhang, 2025). A three-wave study also linked students' ChatGPT use with procrastination and concerns about memory (Abbas et al., 2024). Reviews have described both the potential benefits of AI feedback and explanations and the risks of overreliance and shallow processing (Crompton and Burke, 2023; Farrokhnia et al., 2023; Kasneci et al., 2023; Heung and Chiu, 2025).

AI may support learning when students remain responsible for reasoning, monitoring, and checking. When it replaces these activities, cognitive offloading may result. Intervention and longitudinal studies provide more information about learning processes than cross-sectional self-report studies, but they still mainly assess proximal learning outcomes.

### 4.3 Self-efficacy and motivational regulation in relation to career agency and AI-related insecurity

Research on AI-related self-efficacy and motivation suggests that students' confidence is associated with their AI literacy, learning experiences, and perceptions of their ability to use AI. In higher education, perceived AI ability has been associated with academic self-efficacy and independent learning disposition (Cheung et al., 2025). A quasi-experimental study involving 200 undergraduate STEM students found that AI-assisted writing support was associated with higher intrinsic motivation and self-efficacy, with greater benefits among students with lower academic achievement (Shao and Wang, 2026). Other studies included in this review have also reported associations among AI literacy, AI self-efficacy, motivation, and career commitment (Bewersdorff et al., 2025; Kong et al., 2025; Maulana et al., 2025).

These relationships may operate in both directions. Higher AI self-efficacy or literacy may encourage students to use AI more actively, while successful experiences with AI may, in turn, strengthen their self-efficacy. Prior academic preparation, digital skills, learning motivation, instructor support, and access to AI tools may also influence both AI use and self-efficacy. Existing correlational studies cannot establish the temporal order or direction of these relationships.

AI anxiety and perceived risk of replacement may undermine students' career agency. Studies have reported associations between AI anxiety and AI dependence, less favorable career decision-making, or lower career adaptability (Duan et al., 2026; Wu et al., 2026). Students may experience AI as a resource for practice and learning, while also feeling greater uncertainty about their future. Self-efficacy also needs to be considered alongside demonstrated capability. Students may feel more capable when AI produces fluent content, even if they cannot independently explain or reproduce the reasoning behind it. AI anxiety may affect students' willingness to use AI and their career decisions, but it may also arise from unsatisfactory experiences with AI, lower AI literacy, or concerns about labor-market changes. Cross-sectional studies cannot determine whether anxiety precedes these behaviors, follows them, or changes alongside other factors. Current evidence does not point to a single role for AI-related confidence. Structured practice may support motivation and self-efficacy. Anxiety, dependence, or confidence based mainly on AI-generated outputs may weaken students' independent agency.

### 4.4 Career adaptability as a link between learning and work

Career adaptability has been examined in relation to AI-related learning and career indicators. Research in business and management education has reported associations between AI literacy, perceived AI readiness, and career adaptability (Testa et al., 2026). A study of Chinese university students also found an association between generative AI acceptance and perceived employability, with positive attitudes toward AI and career adaptability potentially contributing to this relationship (Shen et al., 2026). Other studies have examined associations between AI anxiety, career decision-making, and career adaptability (Duan et al., 2026; Wang, 2026).

These findings are based mainly on cross-sectional designs and self-reported measures. They show associations with career-related perceptions or resources, but do not establish whether AI-related learning precedes changes in career adaptability or perceived employability. The direction may also be reversed: students with greater career adaptability may be more likely to explore AI tools and career information. Prior career experience, field of study, institutional support, and labor-market conditions may also influence these relationships. The available evidence therefore does not establish that AI-related learning improves career entry, job quality, or workplace performance. Longitudinal studies following students beyond graduation and measuring employment outcomes directly are needed.

### 4.5 Equity and institutional conditions shaping resource access and capability differences

AI may broaden access to feedback, learning support, and career information, but access to tools does not necessarily lead to equivalent learning gains. Existing studies suggest that prior knowledge, AI literacy, confidence, language resources, access to AI tools, and institutional support may shape how students use AI and what they gain from it (Beckman et al., 2025; Ni and Li, 2026). Students with stronger disciplinary knowledge and self-regulatory skills may be better equipped to evaluate AI-generated outputs, whereas those with weaker foundations may find it more difficult to identify inaccurate information that appears credible. Some studies have found that structured AI support may benefit lower-achieving students (Shao and Wang, 2026), suggesting that AI could help narrow disparities. At the same time, reviews and student-profile studies indicate that the benefits may depend on students' existing resources and the support available to them.

## 5 Proposed framework and educational implications

### 5.1 Proposed pathways and contextual conditions

Figure 3 distinguishes among student–AI engagement, proximal learning and psychological outcomes, career-related indicators, and direct employment outcomes. Solid arrows represent theory-informed directional propositions; the dashed link to employment outcomes marks a proposed translation that remains untested. Dotted connectors indicate contextual moderation, and the return arrow represents a proposed feedback process. The figure should not be interpreted as an established causal or temporal sequence.

**Figure 3:**
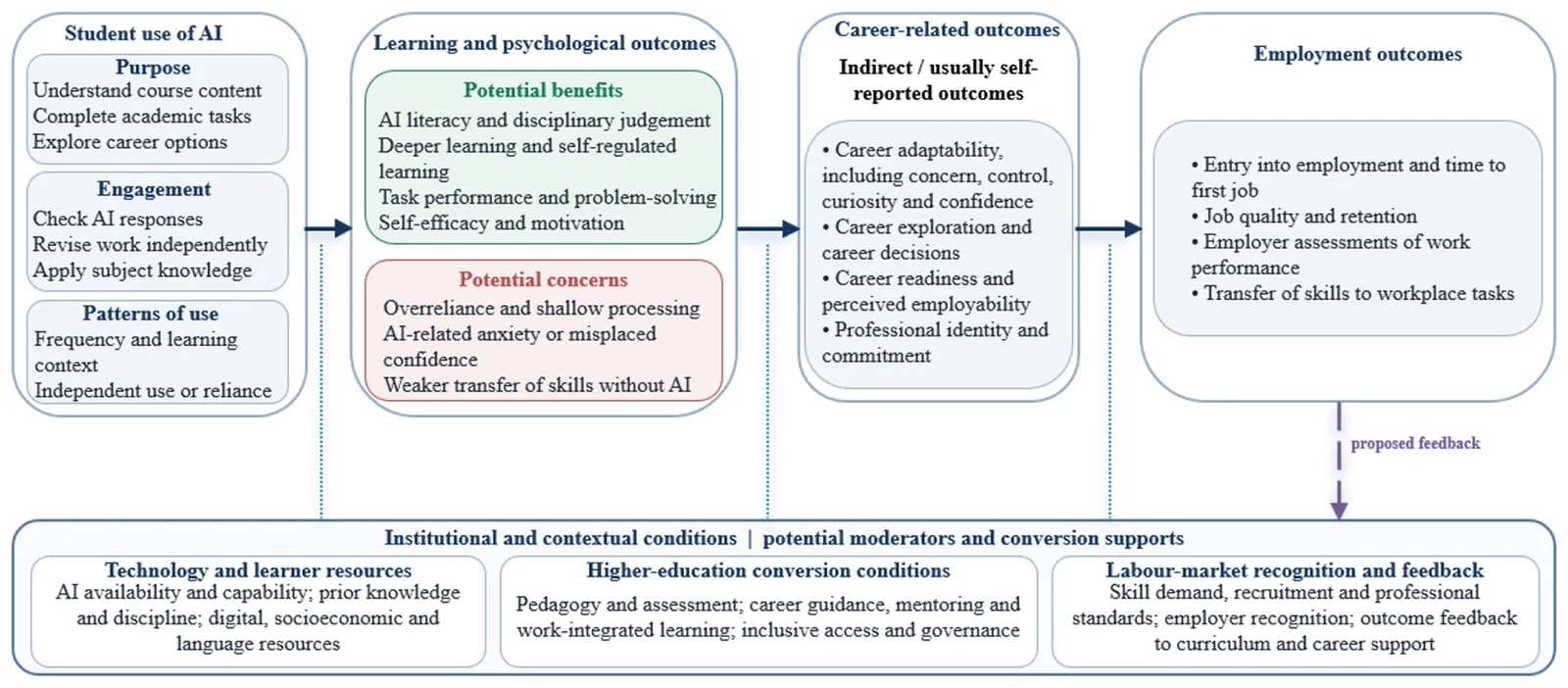
Proposed multilevel pathways linking student–AI engagement with career and employment outcomes, including contextual moderation and proposed feedback.

At the individual level, student–AI engagement varies in purpose, quality, and degree of reliance. Structured use involving independent reasoning, output verification, reflection, and revision may be associated with gains in AI literacy, disciplinary judgement, cognitive engagement, self-regulated learning, and confidence. Substitutive use may accompany cognitive offloading, shallow processing, or anxiety. These relationships may vary with prior competence, task design, and institutional support.

Proximal learning and psychological indicators are distinct from career-related indicators such as career adaptability, career readiness, and perceived employability. Much of the evidence linking these variables is cross-sectional or self-reported. Reverse direction and shared influences including prior competence, discipline, work experience, and institutional support remain plausible. Career-related indicators are also distinct from direct employment outcomes, including employment entry, time to first job, job quality, retention, and employer assessment. Their translation into such outcomes remains a hypothesis because longitudinal follow-up evidence is limited. Graduate and employer outcomes may inform future curricula and career support, although this feedback process also requires empirical testing.

### 5.2 Implications for higher education

AI literacy can be embedded within disciplinary learning rather than delivered mainly through generic tool workshops. Programmes can specify what responsible human AI performance means in their field, what can be delegated, what must be verified, which forms of evidence are required, and which decisions remain the learner's or professional's responsibility. Engineering and computing may emphasize validation, debugging, model limitations, and data reasoning; health and regulated professions require attention to safety, evidence appraisal, privacy, and accountability; humanities and social sciences may focus on source verification, interpretation, argument quality, authorship, and contextual sensitivity.

Assessment design can preserve cognitive agency by asking students to document revisions, verify claims, compare outputs, justify delegation decisions, explain errors, and defend their final work. In foundational courses, staged or partial AI use may be appropriate when students are still developing the knowledge needed to evaluate system outputs. In advanced courses, AI use can be incorporated into authentic tasks while disciplinary judgement remains visible.

A calibration cycle may help distinguish AI-supported performance from independently retained capability: students first attempt a task, use AI to extend or challenge their reasoning, verify the output against disciplinary evidence, and then complete an unassisted explanation or transfer task. Prompt histories, revision traces, verification decisions, and confidence ratings can inform feedback. Assessment research has similarly called for attention to learning processes and responsible AI use (Xia et al., 2024), the DRIVE framework treats AI interactions as possible evidence of learning (Oliveira et al., 2025), while the Artificial Intelligence Assessment Scale aligns permitted AI use with intended learning outcomes (Perkins et al., 2024).

Career education can pair AI-supported occupational exploration with reflection on changing task requirements, transferable capabilities, and alternative career plans. Assisted and unassisted practice, together with comparison of confidence and performance, may help students assess which capabilities they can use independently. Institutions can also monitor differences in access, AI-related confidence and anxiety, disciplinary foundations, and guidance, then provide support responsive to students' needs. These are educational implications of the proposed framework; their effects on employment outcomes require evaluation.

## 6 Discussion

The principal theoretical contribution of this review is to frame the relationship between AI-supported learning and graduate employability as a possible developmental process, rather than as a direct consequence of AI access, acceptance, literacy, or use. The framework proposes several relationships for further investigation. Cognitive engagement and self-regulated learning may be associated with whether AI-supported activity contributes to learning that can be retained beyond the immediate task. Self-efficacy and motivational regulation are included as psychological resources in the proposed framework. Career adaptability may be relevant to how students apply learning resources in career exploration and adjustment. These relationships are proposed pathways; the available evidence does not establish them as causal mechanisms or a confirmed temporal sequence. Broader reviews of higher education employability likewise distinguish educational activities, skill development, and labor-market outcomes (Eimer and Bohndick, 2023; Scandurra et al., 2024).

Recent reviews describe AI literacy as involving more than the operational use of AI systems, including the knowledge and competencies needed to understand and evaluate AI (Almatrafi et al., 2024). Studies included in this review report associations or changes in proximal outcomes such as engagement, self-regulated learning, and task performance following AI-supported activities (Song et al., 2025; Myagmarkhorloo et al., 2026). However, these findings do not establish that AI-supported learning improves later employment outcomes. The literature on AI and self-regulated learning in higher education also identifies both potential support and challenges, indicating that outcomes may depend on the learning task and the guidance provided (Lan and Zhou, 2025). One possible interpretation is that AI may support learning when students retain responsibility for reasoning, monitoring, and checking outputs; this remains a proposition requiring further testing.

Positive perceptions of AI and concerns about its use or employment implications may coexist. Differences in prior competence, perceived control, AI-related anxiety, and perceived occupational change may help explain variation across students; current evidence does not establish a single, uniform effect. Similarly, career adaptability may be relevant to students' career development, but its role in linking AI-supported learning with employability remains uncertain. Studies of AI-related career outcomes have primarily examined career adaptability and perceived employability rather than actual employment outcomes (Shen et al., 2026; Testa et al., 2026). Research on career adaptation among higher education students provides a broader context for considering these relationships, but does not demonstrate an AI-related employment effect (Soares and de Taveira, 2024).

Direct evidence concerning employment status, job quality, or employer evaluations remains limited. Many studies instead measure perceived employability, career readiness, AI literacy, engagement, self-regulated learning, or other psychological and educational outcomes. These measures are relevant to employability research, but they should not be treated as equivalent to actual employment outcomes. Cross-sectional associations also leave the direction of relationships unresolved: for example, students with stronger career adaptability may be more likely to explore AI tools and career information. Prior competence, work experience, discipline, institutional resources, and labor-market conditions may also contribute to observed associations. Evidence about university employees, institutional policies, or implementation should therefore be interpreted as contextual evidence, rather than as direct evidence about student-level learning or graduate employment. Employment outcomes are shaped by more than students' learning experiences and psychological resources. Recruitment and workplace performance may also depend on disciplinary competence, communication, professional presentation, punctuality, teamwork, collaboration, leadership, prior experience, and employer expectations. These factors were not consistently measured in the studies reviewed. Positive self-reports of readiness or confidence therefore cannot establish that graduates demonstrate these competencies in recruitment or workplace settings.

The evidence base was unevenly distributed across publication years, countries, disciplines, and study designs. Of the 46 included publications, 24 were conducted in mainland China and 41 were published in 2025 or 2026. Classified by their primary study context, 26 were broad or multidisciplinary, or did not specify a discipline; eight focused on business or management; three on vocational or applied education; three on language-related fields; two each on nursing and STEM or engineering; and one each on teacher education and humanities or social sciences. Methodologically, 28 studies used cross-sectional surveys, 12 used intervention, quasi-experimental, or longitudinal designs, four were qualitative studies, and two analyzed documents or employment-related data. This concentration of recent research, Chinese settings, and cross-sectional surveys limits the transferability of findings across disciplines and national contexts. Only a small subset of studies reported employment status or employment rates, and only one followed graduates' employment status 6 months after a course.

Findings were not uniformly positive. AI anxiety was not significantly associated with career decidedness among students in English language teaching and English language and literature, although a weak negative association was found among translation and interpreting students (Yaşar and Karagucuk, 2025). AI anxiety did not significantly predict lifelong learning tendency among pre-service teachers, and self-efficacy did not significantly moderate the relationship between AI anxiety and career decisions in another study (Cantaş et al., 2026; Duan et al., 2026). Other studies found no significant direct relationship between generative AI learning support and employability, or between continued intention to use AI and career-choice intention (Cheng et al., 2026; Yuqian et al., 2026). Results may therefore vary with discipline, task, prior achievement, and institutional context. For example, structured AI-assisted writing appeared to benefit lower-achieving students, whereas another study associated stronger prior quantitative skills with greater AI capital (Drydakis, 2025; Shao and Wang, 2026). These findings suggest that prior competence, field of study, and institutional support may be relevant boundary conditions. Evidence concerning socioeconomic position, disability, language proficiency, and access to paid AI tools remains limited.

Future research should examine these proposed relationships over time. Longitudinal studies could follow students from AI-supported learning experiences through career preparation and into employment, measuring both intermediate outcomes and subsequent labor-market outcomes. Experimental and quasi-experimental studies could compare learning activities that require students to verify AI outputs, explain revisions, or complete later tasks without AI assistance. Interaction logs, revision histories, confidence accuracy calibration, independent error detection, and delayed transfer tasks may help distinguish perceived fluency from independently demonstrated capability. Studies should also account for alternative explanations, including prior achievement, work experience, discipline, institutional support, and labor-market conditions. Where feasible, outcomes should include career entry, job quality, employer assessments, and performance on authentic professional tasks. Cross-disciplinary and cross-cultural comparisons may clarify how professional norms, access to AI, and employment conditions shape these relationships.

### 6.1 Limitations

Several limitations qualify this review. Its critical narrative design prioritized conceptual integration and did not include formal risk-of-bias scoring or quantitative effect estimation. The search was restricted to English-language publications indexed in Web of Science, Scopus, ERIC, and PubMed. Relevant studies in other languages or outside these databases may therefore have been missed. Backward citation tracking helped identify foundational work but may have favored established citation networks. Gray literature was not systematically searched, and publication bias could not be formally assessed. The evidence base is also changing rapidly, with 41 of the 46 included publications appearing in 2025 or 2026; future research may alter the current interpretation. Finally, direct evidence linking AI-supported learning to graduate employment remains limited, while employment outcomes are also shaped by economic, organizational, and labor-market conditions beyond the individual learning and psychological processes examined here.

## 7 Conclusion

AI adoption rates, prompt proficiency, and short-term task performance alone provide an incomplete account of AI's role in higher education. This review identifies cognitive engagement, self-regulated learning, self-efficacy, and career adaptability as learning-related and career-related resources that may be relevant to graduate employability. However, the available evidence, which mainly concerns proximal learning outcomes and self-reported career indicators, does not establish that AI-supported learning improves employment rates, job quality, or workplace performance.

The framework presents possible relationships for future investigation rather than an established causal or developmental sequence. AI-supported learning may contribute to resources relevant to employability when students retain responsibility for reasoning, monitoring, and verifying information and receive appropriate pedagogical and institutional support. Whether these resources translate into employment advantages requires longitudinal research that follows students into the labor market and measures outcomes such as career entry, job quality, skills transfer, and employer evaluations. Such research should also consider disciplinary competence, communication, teamwork, leadership, and other workplace expectations alongside students' learning and psychological outcomes.
